# Elements of Medicine

**Published:** 1855-12

**Authors:** 


Art. VI.—*Elements of Medicine: A Compendium View of Pathology and Therapeutics; or, the History and Treatment of Diseases.* By Samuel Henry Dickson, M. D., LL. D., Professor of the Institutes and Practice of Physic in the Medical College of the State of South Carolina. 8vo. pp. 752. Philadelphia: Blanchard & Lea. 1855.

Prof. Dickson, the author of the above work, has long occupied a very prominent position in the profession of this country. For thirty years, as he informs us in the preface, he has been a teacher of medicine, and a student for more than forty. He has prepared and published many manuals or text-books for those in attendance upon his lectures; and in addition has written and printed several volumes upon medical subjects in general. The "Elements of Medicine" are the product of a careful collection of what has been regarded most valuable in both, with such matter as continued study and enlarged experience have enabled him to add.

The work is divided into two parts, the first being devoted to General Pathology, and the second to Pathology and Therapeutics. Under the head of General Pathology, the Nature, Etiology, Seats, Phenomena, and Tendency of Diseases, are discussed at considerable length. This portion of the Elements abounds in interesting facts, and is, in our opinion, the more valuable half of the book. We feel obliged, however, to say just here that this contains statements which are not in accordance with the views of many of the best minds of the profession, and a number of paragraphs devoted to speculation and disputation which we think might have been omitted altogether without loss, or the space they occupy given with advantage to pathology proper and therapeutics. In order that we may do entire justice to our subject, we may substantiate at least the greater part of what is contained in the paragraph just written by Dr. Dickson's own testimony. "The *tendency*," he says, "of all the various forms of *disease* is essentially and in their own nature to death; death either of a part or of the whole body, according as the morbid affection has been general or local." Our author thinks that this opinion is likely to startle the believers in the ancient doctrine of the *vis medicatrix naturæ*—and we agree with him. The multiplied forms of disease are but the common result of certain causes or circumstances which when brought into contact with the animal body obstruct its regular and natural actions. The removal, or change or cessation of these causes is, according to Prof. Dickson, entirely adequate to the explanation of recovery from any morbid state whether general or local without its being necessary to resort to the theory of the curative forces of nature.

Fortunately these causative agencies are often transient in their application, influence, and effects, or are removed by some change of circumstances; and ceasing to act, the disease produced by them necessarily comes to an end, and the train of natural and healthy movement goes on again; but if the influence of these causes continues permanent, the disease will continue. Disease is not "the product of any spontaneous action of any organ or system of parts, or of any of the vital forces or causes; neither can any separate natural provision be shown to be made for its removal or arrest, and most surely not, as has been imagined, by the very processes in which it consists."

The natural tendency of disease being stated, the reason why its termination is not uniform is found in the fact that the cause which produces it is not uniform. This cause, for instance, "is originally somewhat less than mortal in its intensity, or its force undergoes diminution; the excitability on which it first acted wears out; the predisposition to which it was adapted is altered in some mode; the system becomes habituated and thus callous to its irritation—as a ball will sometimes remain quietly lodged in a part which it had inflamed severely; owing to some one or more of these conditions, all amounting virtually to a removal of the cause of disease, it comes to an end (*causa sublata tollitur effectus*) before its natural tendencies are manifested in their ultimate result—before either death or disorganization has occurred. It is thus we explain the apparently spontaneous restoration of health every day met with, and so apt to be attributed to the *vis medicatrix*—the restorative energies of the constitution." And Dr. Dickson acts upon his theory by refusing to trust to the capacity for resistance in a disordered and deranged system. He holds that "the office of the physician is to remove, if possible, the source of evil; to counteract its influences, and to diminish their intensity by every means in his power; to subdue the predisposing excitability upon which it is fitted to act, and has acted; to change by revulsive interference the determination of its agency, and save a vital organ or a delicate portion of the animal structure, by derivation to a stronger or less important part."

But we cannot dwell longer on this portion of the Elements. The chapter on Fevers is one of the best in the work, and fully sustains the reputation of the distinguished author We are sorry that we cannot use the same terms in regard to the section devoted to Diseases of the Respiratory System. It is in this section that Dr. Dickson's work is most unsatisfactory. Describing the physical signs of bronchitis—which disease in its acute form occupies but little over a page of the work—he says: "Resonance upon percussion is dull, and the respiratory murmur is impaired very generally over the chest." Surely Dr. Dickson did not intend to say that, as a general rule, there was dullness on percussion in bronchitis. He must know that the percussion-sound, as a rule, is not impaired in clearness, and may even be a little raised above the pitch of health. In view of the turgescence and thickening of the mucous membrane being among the more prominent of the anatomical features of the disease, the usual clearness of resonance is, it will be admitted, a matter of no little surprise. But it is not the less a fact, and a most important one it is. Nor is *impaired* the word which correctly and best describes the change on the part of the respiratory murmurs in this affection. For while these are weakened—and only in so far impaired—occasionally, even suppressed, in the tissue about the inflamed tubes, they are exaggerated on its confines and elsewhere—hence especially so in the upper parts of the chest. No mention is made of either the sonorous, sibilant, or mucous ronchi.

The diagnosis of chronic bronchitis is disposed of as follows:—

"The distinction between chronic bronchitis and tubercular phthisis is often difficult. In the latter there is less crepitus or râle, less soreness of the trachea and thorax, more tendency generally to hemoptysis, and less expectoration in the early stages. In their advanced progress we can draw no line between them except their previous history."

Dr. Dickson says in his preface, that while "it was necessary that the book should be as compendious as possible, it was also absolutely necessary that nothing essential to a fair development of the whole subject attempted should be left out." We certainly have no fault to find with the compendious character of the above description of chronic bronchitis. The most impatient student when hardest pressed for time could hardly, we think, fail to be satisfied with its brevity. But short as it is, we may ask is it free from errors? Walshe states that the physical signs of chronic bronchitis are in the main those of the acute disease, and surely these do not so closely resemble those of pulmonary consumption that no line can be drawn between them. If our experience or reading is to go for anything, the only form of this affection in which any great difficulty in distinguishing it from tubercle is likely to arise, is in that condition familiarly known as *dilatation of the bronchi.* This state, but little dissimilar to that of phthisis, may coexist with physical signs, so resembling those of a cavern, that it is sometimes impossible to affirm whether a given case be one of tubercle with excavation, or one of globularly-dilated bronchus with surrounding consolidation. Louis, in his work on Phthisis, reports a case of this kind. According to the best authorities on chest disease, it is, however, only this form of bronchitis, or the compound state where bronchial dilatation and tuberculous excavation coexist—both of which conditions are very seldom seen—that are beyond the reach of diagnosis.

Pleuritis, which is only considered in its acute form, is described by Prof. D. as coming "on with sharp pain in the thorax." Are not most attacks of pleurisy observed to commence with rigors, these being actually the first symptom, and precede the "stitch in the side" in many cases by some hours or even days? "Fever soon supervenes, with quick, frequent, hard, and full pulse; the skin is hot and dry, and cough comes on," etc. No one will deny that the general symptoms of acute pleurisy are those of a febrile inflammation. The pulse, as Dr. Dickson remarks, is quick and frequent; but are there not a great many cases in which it is not hard? The skin is hot but not burningly so, even at the outset, and at the period of effusion, according to Walshe, it nearly always becomes moist.

In relation to the prognosis of this affection, our author states that "in *general* it is a manageable disease, attended with little danger, if the patient is seen *early.*" [The italics are our own.] We understand his remarks to apply to pure, primary, idiopathic pleurisy. Now death is so rare a result of this kind of pleurisy with or without effusion, when occurring in individuals free from organic complications, that Walshe and Louis affirm they have never lost a patient from it nor known an occurrence of the kind in the practice of others.

Pneumonia is the next disease in order. The physical signs of this affection our author regards of "great importance to be attended to," and yet they are allowed to occupy but eleven lines. We do not complain of the succinctness of the description, but we must be allowed to call attention to at least one grave error found in the following lines: "When purulent matter is spit up, we hear the mucous râle, or a gurgling; there is restored resonance on percussion, if a cativity is formed pectoriloquy will show itself." We will not stop to discuss the question of the number of cases in which this mucous râle is never heard, and yet which unquestionably are examples of pulmonary suppuration, but will merely ask, did any reader of this article ever know the percussion sound to become normal over the seat of the disease during the progress of interstitial suppuration of the lung? "Restored resonance on percussion" is at no time a mark of pneumonic suppuration. There are only two conditions of the lung in pneumonia in which the sputa are purulent; these are intestinal suppuration where the suppurating points communicate with the bronchi, and pulmonary abscess, of which the contents are more or less completely evacuated. In the former of these states the physical signs are in the main those of red hepatization—dullness upon percussion being seldom wanting. In the latter, the percussion-sound is dull, and the parietal resistance marked; though in rare cases the resonance is tubular, amphoric, or cracked-metal. Pectoriloquy will show itself only when the abscess is superficially situated and empty. In the case of an abscess, with its contents retained, there are no signs of an absolutely distinctive kind. The vocal resonance may be strongly broncophonic, nothing more, and this might have existed long before the formation of an abscess.

Concerning the manner in which our author treats diseases we do not think it necessary to express an opinion. Dr. Dickson is a man of very extensive observation; he is reputed to be a gentleman of nice discrimination and excellent judgment, and in his native State and throughout the South is esteemed one of our best practitioners. What he has written on therapeutics is, of course, the result of his experience which has been ample. Remembering what his tenets are relative to the tendency of disease, it is easy to foresee what would be the general character of his medication. The stress which he lays upon blood-letting in the treatment of pneumonia is so widely opposed to the prevailing professional sentiment in the South on this subject that we will quote that portion of his remarks which refer to the lancet:—

"Venesection is a remedy specially adapted to the contingencies of this formidable disease, as it usually presents itself; whose importance is scarcely exaggerated by Bouillaud, greatly underrated by Louis, and by no means sufficiently dwelt on even by Bartlett, in his elegant *Essay on the Certainty of Medicine.* In the early stages—in the first, second, third, or perhaps the fourth day, it can scarcely ever be proper to omit it. . . . . . . . . The breath should be more freely drawn, the pain should be lessened, or the pulse brought down nearly to the point of syncope before the arm is tied up. If the subject be young and robust, the bleeding may be repeated as soon as the pain or dyspnœa return, perhaps twice or thrice in the twenty-four hours. I am surprised at the hesitating manner in which a large proportion of modern writers speak of the effect of this measure. I am very confident that I have many times thus arrested the disease in its forming stage, and that my patient has at once begun to convalesce. This, however, will only happen when he is seen within the first few hours. . . . . . . . . . But at any period while there is pain and dyspnœa, with good pulse and strength, the lancet may be employed with advantage. It gives almost unfailing relief in greater or less degree, and is highly useful too in preparing the system to receive the benefit of the farther necessary treatment. I need not say that cases are met with in which it would certainly do more harm than good. In advanced life or early infancy, in greatly debilitated subjects, when time has been lost and the strength impaired, when the associate fever is of low type, or the case is secondary, and the subject exhausted by previous disease, it must be abstained from."

For the treatment by emetic tartar our author prefers "to substitute other sedatives, relaxants, and diaphoretics, whose action is less irritating and safer." To eight ounces of a strong infusion of serpentaria or seneka, he adds one drachm of ipecac and thirty drops of laudanum. Of this mixture he directs the patient to take a tablespoonful every half hour till nausea is produced. Whatever we in this latitude may think of our author's heroism in the abstraction of blood, we can but allow that he is very mild with opium. Two drops of laudanum every half hour is gentle enough for the gentlest. In another paragraph, however, he thus speaks of the great hypnotic remedy:—

"Opium is a remedy which I am never able to dispense with. From the very beginning, I prescribe it in free doses at night, giving to an adult, of Dover's powder from ten to fifteen grains at bedtime, to be repeated at midnight if restless. Throughout the day I combine it in smaller quantity with whatever other recipe he is taking. It is our best demulcent, diaphoretic, sedative, and tranquilizer. It is contraindicated only in the rare complications of cerebral disorder and congestion."

Our author is persuaded that he has seen the veratrum-viride, as he has seen the lancet, cut short the disease abruptly and cause it to abort. Of this we think from the assurances that we have received from many quarters there can be but little doubt. We ourselves have known the veratrum to exert the most powerful influence upon pneumonic congestion.

Chronic pneumonia—*apostematous phthisis*—is next considered, and we are obliged to say in not a very satisfactory manner. Chronic pneumonia is *not* a frequent sequel of the acute disease, is rare as a primary disease, and is common only as a local complication in tubercle, cancer, and other adventitious products in the lung. Chronic pneumonia, properly so called, manifests no tendency to softening. These, at least, are the views of the best writers on pulmonary diseases, and differ in every respect from those expressed by our author. We very much doubt whether the chronic pneumonia described by Dr. Dickson ever supervenes upon asthma, catarrhal fever, or bronchitis. And since this disease, either in the form described by writers on lesions of the respiratory organs, or as (we must think incorrectly) delineated by our author, is an exceedingly rare affection, would it not have been better to have omitted all mention of this form of pneumonia and described the inter-current variety—the importance of which cannot easily be exaggerated—and which, in truth, constitutes the bulk of the cases of this affection which are encountered in practice?

The prognosis, our author states, is "rather unfavorable." Is it not, on the contrary, exceedingly so? "The duration varies from a few weeks to as many months; in the former contingency it is spoken of by the vulgar as a 'galloping consumption.'" We regret being obliged so often to take issue with our author, but we cannot allow so obvious an error as is contained in the lattter part of the above sentence to go unnoticed. Is it true that the vulgar call that form of chronic pneumonia which terminates acutely, "galloping consumption?" Acute phthisis, we admit, is not at all unlike pneumonia in many respects. But acute phthisis is *not* chronic pneumonia, though the latter disease were to end fatally in three weeks. Chronic pneumonia and galloping consumption are by no means convertible terms.

Tubercular phthisis is next treated of. Quoting from Theophilus Thompson our author says: "The wavy inspiration probably indicates the presence of some deposit of impaired vitality." . . . "Walshe lays the same stress upon the prolongation of the expiratory murmur." Prof. Dickson is mistaken in respect to the sentiments of the latter; the following is the language held by Dr. Walshe in regard to this sign: "Prolonged expiration, if unattended with alteration of quality, is insignificant; under such conditions it is in all probability a normal state: and even coupled with slight harshness and coarseness of quality, it must be cautiously received as evidence in females, and at the right side."

Again, Dr. Dickson, speaking of the physical signs of the first stage of phthisis—and to this entire subject he devotes but ten lines—says "there may be dullness just under the clavicle on percussion." We submit that the language used by our author does not convey anything like a proper idea of the percussion sound in the first stage of tubercular phthisis. Again: "When softening occurs, the surrounding inflammation consolidates the tissue, and we have loss of vesicular murmur and morbid dullness." And is this all? Is there no dullness of percussion till softening sets in? Is there no loss of vesicular sound till inflammation solidifies the surrounding lung substance? Is not the respiratory murmur often deficient, nay, wholly absent, in many instances before suppuration occurs? Do not both the percussion and vesicular sounds frequently present the characters just attributed to them in the first stage of phthisis—a period wholly distinct and separate from that described by our author? It must be admitted that they do.

Once more and we have done with the ungracious task of pointing out errors, some of which, we are sure, must have occurred through inadvertence: "When a vomica is formed, and a cavity is more or less emptied, we have resonance and pectoriloquy; to which are added the metallic tinkling, and when the cavity grows large, amphoric resonance." This is far from being clear, and is not quite correct. It is only where a considerable stratum of healthy parenchyma lies between the excavation and walls of the chest that resonance on percussion will be elicited over tuberculous cavities; and even here the blows must be gentle. Strong percussion will yield moderate dullness in spite of the intervention of a very great amount of normal vesicular structure. And, on the other hand, if the cavity be small, and surrounded by much consolidation, the percussion sound will be absolutely dull, or even tubular. Or, again, should there be several small excavations with indurated lung-substance existing between, percussion is unquestionably dull, to say nothing more.

Auscultators are almost uniformly agreed that there are no physical phenomena so fallacious as those furnished by the voice; and of all forms of vocal resonance none are so unreliable as pectoriloquy. From the position which it occupies in the quotation just made, we might infer that our author attaches to it a very different significance. The true value of this change on the part of the voice is indeed singularly trifling. In cavities in the lungs the voice may be either natural, weak, null, amphoric, broncophonic, or pectoriloquous. We have always been taught, and our experience at the bed-side has confirmed the opinion, that if there be any voice sound which can take rank as distinctive of an excavation the only one is *whispering* pectoriloquy; and cavities may exist without even this. No one can have paid much attention to physical diagnosis without having heard broncophony quite as often as pectoriloquy in pulmonary excavations.

But we must bring our notice of Prof. Dickson's 'Elements' to a close. Of very many of the views entertained by the learned author we did not propose in the outset to express an opinion. We have taken issue with him on no point of therapeutics. As a practitioner of large and varied experience, and of the highest reputation in the region of country where he has encountered disease, his views in respect to practice are entitled to great consideration. Where we might have been tempted to differ with him on these subjects, we have felt that it would not be seemly in us, in Kentucky, to sit in judgment upon the practice of a brother in South Carolina. Climate and habits are elements in the constitution of disease which must not be disregarded. Where we have ventured to find fault with the work, it has been upon points of diagnosis and especially those pertaining to the physical branch of this subject, in regard to which geographical lines exert no influence. In a future edition, which, we are sure will be called for, the distinguished author will undoubtedly remove these blemishes, many of which are evidently the result of oversight.

